# 25 MHz clock continuous-variable quantum key distribution system over 50 km fiber channel

**DOI:** 10.1038/srep14607

**Published:** 2015-09-30

**Authors:** Chao Wang, Duan Huang, Peng Huang, Dakai Lin, Jinye Peng, Guihua Zeng

**Affiliations:** 1 State Key Laboratory of Advanced Optical Communication Systems and Networks, Shanghai Key Laboratory on Navigation and Location-based Service, and Center of Quantum Information Sensing and Processing, Shanghai Jiao Tong University, Shanghai 200240, China; 2 College of Information Science and Technology, Northwest University, Xi’an 710127, Shanxi, China

## Abstract

In this paper, a practical continuous-variable quantum key distribution system is developed and it runs in the real-world conditions with 25 MHz clock rate. To reach high-rate, we have employed a homodyne detector with maximal bandwidth to 300 MHz and an optimal high-efficiency error reconciliation algorithm with processing speed up to 25 Mbps. To optimize the stability of the system, several key techniques are developed, which include a novel phase compensation algorithm, a polarization feedback algorithm, and related stability method on the modulators. Practically, our system is tested for more than 12 hours with a final secret key rate of 52 kbps over 50 km transmission distance, which is the highest rate so far in such distance. Our system may pave the road for practical broadband secure quantum communication with continuous variables in the commercial conditions.

Quantum key distribution (QKD) is able to enable two remote participants to share unconditionally secure keys based on the laws of quantum mechanics^1^,^2^,^3^. By combining with one-time pad encryption, the QKD is promising for effectively ending the cat and mouse game between the guardians of secrets and their enemies^4^ and it has now become one of the most dynamic research fields. Since the first experimental demonstration of the QKD over 32-cm free-space channel in 1992^5^, tremendous efforts have been made to keep pace with the demands in large-scale and broadband secure quantum communication^6^,^7^,^8^,^9^,^10^,^11^. The QKD technologies are also being considered in practical applications^12^.

In the past decade, the continuous-variable QKD (CVQKD) has been explored as an alternative approach to achieve possibly higher secret key rates^13^,^14^,^15^. After the first experiment has demonstrated the QKD protocol with the Gaussian-modulated coherent states (GMCS)^16^,^17^, the CVQKD has shown its potential advantages and its ability has been proven on being secure against general collective attacks^18^,^19^,^20^ and coherent attacks^21^,^22^,^23^. Currently, the GMCS protocol has become a main way for the CVQKD experiment over telecommunication fibers. For example, Qi *et al.* presented an experimental scheme to obtain a secure key rate of 0.3 bit/pulse over a 5 km fiber link^24^. Xuan *et al.* reported a CVQKD experiment with final secure key rate of 3.45 kbps over a distance of 24.2 km of optical fiber^25^. Dai *et al.* implemented a CVQKD system over 27.3 km optical fiber channel and achieved 8.6 kbps secret key rate^26^. Fossier *et al.* realized the field test for the CVQKD system at 500 kHz repetition rate over 15 km fiber channel and generated an 8 kbps final secret key rate^27^. And Jouguet *et al.* demonstrated a stable CVQKD system which can run about six months with a low secret key rate^28^. Especially, with the help of multidimensional reconciliation protocol^29^, Jouguet *et al.* experimentally demonstrated that the CVQKD can be implemented at a distance more than 80 km^30^.

Unfortunately, all the clock repetition rates of the most existing experiments are not higher than 2 MHz and this imperfection leads to the low secret key rate. For practical application, it is very important to pursue higher secret key rates with longer secure distances. In this paper, a practical CVQKD system is developed and it can run stably in a commercial fiber network at a 25 MHz clock repetition rate and output a final secret key rate about 52 kbps over 50 km fiber channel. Our system is implemented based on the well-known GMCS QKD protocol^17^, and several novel techniques are developed, including the broadband homodyne detection, precise phase compensation, and high-rate multidimensional reconciliation. It is worth noting that this paper has presented a practical CVQKD system with highest secret key rate at the secure distance of 50 km over a standard telecom fiber, thus our CVQKD system may pave the road for broadband secure quantum communication with continuous variables in practical applications.

## Results

### The 25 MHz CVQKD system

**Figure 1:**
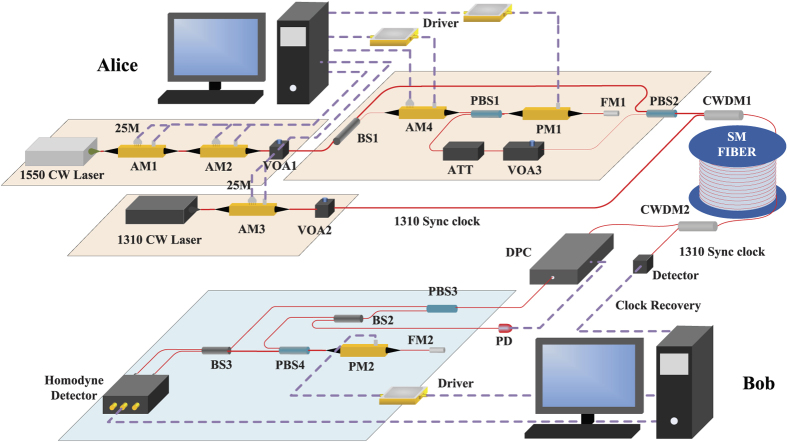
The layout of 25 MHz CVQKD system. CW Laser, continuous wave laser; AM, amplitude modulator; BS, beam splitter; PM, phase modulator; PBS, polarizing beam splitter; FM, Faraday mirror; CWDM, Coarse Wavelength Division Multiplexer; ATT., attenuator; VOA, Variable Optical Attenuator; DPC, dynamic polarization controller. C.W. draws the whole Fig. 1.

Our system consists of three key parts: optical module, controlling module and post-processing module. The optical module is employed to implement encoding and decoding of secret key by using quantum signal states and local oscillator (LO) signals. The optical module also includes synchronization and classical communication which are necessary in a CVQKD system. The controlling module is used to support the optical module so that the classical and quantum communication procedures can run well. The post-processing procedures module is employed to implement error correction and privacy amplification to output a final secure key. The layout of our proposed CVQKD system is plotted in Fig. 1.

In the optical module, our experiments are performed based on the GMCS QKD protocol. The sender, Alice, generates a GMCS and sends them to the receiver, Bob, who measures one of the quadratures of these states using a broadband homodyne detector. Practically, a continuous-wave output from a 1,550.12-nm wavelength laser is transformed into a 25 MHz coherent pulse train by two amplitude modulators, where some pulses are randomly chosen for security monitoring. Then these pulses are split into a strong LO path and a weak quantum signal path. The key information is encoded in the quadratures of amplitude and phase of the coherent optical pulses according to a centered Gaussian distribution. By using the polarization-multiplexing and time-multiplexing techniques, the quantum signal together with the LO signal are sent to Bob through a 50 km fiber link. In the receiver’s side, Bob randomly measures one of the quadratures with a 300 MHz bandwidth homodyne detector and outputs the results with a 1 GS/s data acquisition module. In addition, a coarse wavelength division multiplexer (CWDM) is used to integrate the signals for classical communication, which includes a 1310 nm wavelength clock synchronization signal and two independent transmit-receive classical signals with 1410 nm and 1430 nm wavelength used for reconciliation. All these classical signals are transmitted through a fiber link with the quantum signal and LO simultaneously.

To guarantee the system running at the 25 MHz clock repetition rate, a broadband homodyne detector is developed^31^. In the employed detector, we use a new method to effectively enhance the bandwidth of a detector while still reserving the advantage of shot noise to the electronic noise ratio. In this way, the detector can work stably to get a 14 dB gain between shot noise and electronic noise when running at 25 MHz. In our system, the involved electronic noise and the detection efficiency of the proposed homodyne detector are 0.05 and 60.141%, respectively. Here, it is worth noting that the electronic noise *v*_*el*_ = 0.05*N*_0_ is very high in our high-speed homodyne detection. Fortunately, it is sufficient to achieve a secret key with such a high level of electronic noise in this work.

The controlling module performs three functions in our system, including quantum signal modulation, quantum signal transmission synchronization, and measurement data collections. All these are performed in Industrial Personal Computer (IPC) in which a clock board and field programmable gate array (FPGA) board are used. To modulate the quantum states, two random variables, which satisfy the Rayleigh distribution and the uniform distribution are produced at Alice’s side by a quantum true random-number generator to modulate on the AM4 and PM1. While at Bob’s side, the random variables satisfying the uniform distribution which are produced by quantum true random-number generator are modulated on the PM2 to randomly select *X* or *P*. The measurement results of homodyne detector are collected using a high speed data acquisition (DAQ) board. To implement the transmission synchronization of the quantum signals, a clock pulse signal with 25 MHz repetition rate and 50% duty cycle is generated. The clock signal for synchronization is detected and recovered by Bob, which is used as the synchronization input for FPGA board in IPC at Bob’s side.

In the post-processing module, the important issues are the efficiency and speed of the reconciliation algorithm which are related to the error correction code (see Section: Methods). In our system, to match the 25 MHz clock repetition rate, an optimal LDPC code for the reconciliation procedures is employed to promote the speed and efficiency. In the involved LDPC code, we use vectorized code to diminish the complexity of decoding, and further improve the performance with parallel processing technique. The reconciliation efficiency with such LDPC code can reach 96.9%, and the complexity of decoding is decreased from 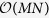 to 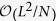 (see Section: Methods), where *L* is the number of element 1 in H, while *M* and *N* are column and row number of H matrix, respectively. Note that *H* is a sparse matrix, *L*^2^/*N* is generally less than 0.01 · (*M* × *N*). Practically, a 25 Mbps decoder is achieved with this code with the help of a GPU device which is employed in our CVQKD system.

### The System Stability Optimization

The stability is an important issue for a practical CVQKD system. Generally, many factors, such as the phase drift, polarization drift, and imperfection of modulators, may influence the stability of the system. These influences may not only relate to the stability but also reduce the final secret key rate. Accordingly, three methods are developed to improve the stability of the CVQKD system, which include a polarization feedback algorithm, a phase compensation algorithm and a related stability method on the modulators.

First, we consider the polarization drift. The polarization drift will become severe for long-distance transmission across optical fiber. In order to ensure long-term stability without manual intervention on the CVQKD system, a polarization feedback algorithm is proposed (see Section: Methods). According to this feedback algorithm, an experiment is performed for controlling the polarization stability. In this experiment, the mean value of the voltage is *μ*_*pola*_ = 0.7391 and the standard deviation is *σ*_*pola*_ = 0.2215 before the use of the feedback algorithm, this gives the jitter ratio *r* = 29.97% of the polarization drift. After the feedback control, the experimental results show that the mean value is *μ*_*pola*_ = 0.9945 and the standard deviation is *σ*_*pola*_ = 0.0021, indicating that the jitter ratio is *r* = 0.21%, which meets the requirement for our experiment.

**Figure 2:**
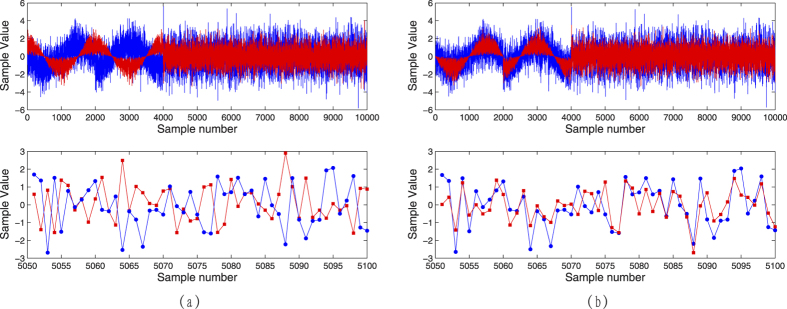
Diagram of the phase compensation with *SNR* = 0.9. The red points indicate the transmitted data at Alice’s side, and the blue points indicate the received data at Bob’s side. (**a**) The uncompensated waveform, (**b**) The compensated waveform.

Now we consider the phase drift in the CVQKD system, which is inevitable due to variation of temperature and vibrations in the environment. The phase shifts may lead to low probability of error correction. Since the reconciliation algorithm could recover only the original signal from the signal which overlaid by addictive white Gaussian noise (AWGN), the phase shifts will hinder the data reconciliation process, resulting in null secret key rate. To overcome the phase drift in low signal-to-noise ratio condition, we propose a simple phase compensation algorithm to estimate the value of phase drift (see Methods). Making use of this algorithm, the continuous variables shared by Alice and Bob have few discrepancies on phase and linear scale. The main difference, which can be solved by reconciliation, is that Bob’s data is overlaid by AWGN. We test the phase compensation in practice, the results are shown in Fig. 2. The precision can reach 0.1° for each frame, which quite eliminates the excess noise from phase shift.

Except for the polarization drift and phase drift, the stability of the bias voltages applied on the AMs for preparing optical pulses will also influence the CVQKD system. This is because the bias voltages will drift over time so that the magnitude of optical pulses will vary in time. In order to stabilize the output pulses, we use an automatic compensation module of bias voltage to perform the feedback control. The compensation module is connected to the optical link and splits a small portion of light for feedback detection. This generates a well stabilized effect on the generation of optical pulses sequences. In practice, the variation of the environmental factors, such as mechanical vibration, temperature and humidity, will also deteriorate the secret key rate and stability. These factors have been optimized technically in our system.

### The Security and Performance

We firstly consider the security of the proposed system. Our CVQKD system is implemented based on the GMCS QKD protocol, theoretical security of the involved protocol has been confirmed even when considering the finite-size effect. Thus the main secure issues are on the practical security of the proposed CVQKD system. Recently, some typical attack strategies have been proposed, such as the wavelength attack^32^,^33^, LO fluctuation attack^34^ and calibration attack^35^. Fortunately, all this attacks can be defeated by adding wavelength filters before Bob’s detector, monitoring the power of LO and using an extra homodyne detector to monitor the real-time shot noise, respectively. Therefore, our system is secure against the presented practical attack strategies.

Next we consider the performance of our CVQKD system. Due to the finite-size effect^19^, if the sampling data for parameter estimation are not sufficient, one cannot precisely estimate the parameters of quantum channel. Subsequently, higher secret key rate and longer secure distance cannot be achieved. In our system, we adopt a block size of 10^9^ and a repetition clock rate of 25 MHz. In this case, considering the parameter estimation and the frame overhead, each block costs only about 2.6 min for data sampling, which is optimal in practical implementation. Then, we evaluate the secret key rate with the finite-size effect under the collective attack, which can be expressed as,


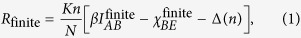


where 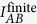 denotes the Shannon mutual information between Alice and Bob, 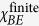 is the Holevo bound defining the maximum information available to Eve on Bob’s key, and Δ(*n*) relates to the security of privacy amplification with the form 
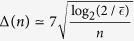
 for *n*≥10^4^. The involved parameters are the modulation frequency *K*, number of the total exchanged signals *N*, the number of the signals for key establishment *n*, the reconciliation efficiency *β*, and the smoothing parameter 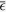, which is the error probability during the privacy amplification.

**Figure 3:**
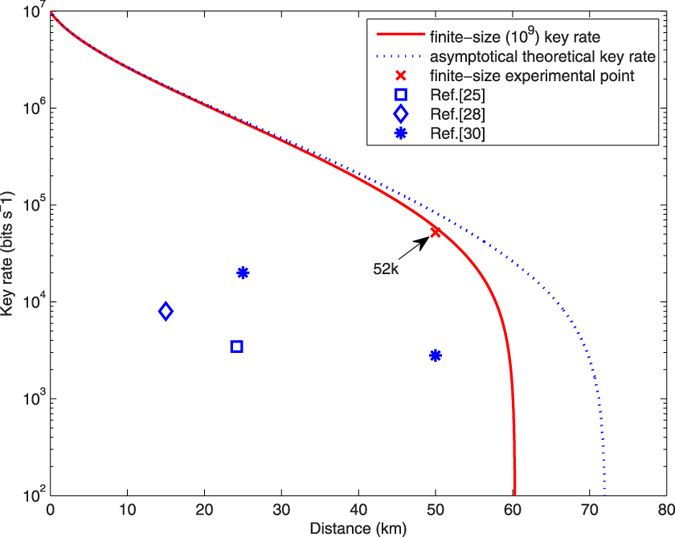
The final secret key rate of the 25 MHz CVQKD system. The blue dash line denotes the asymptotical theoretical key rate, and the red solid line is the finite size theoretical key rate with block size of 10^9^. The modulation variance *V*_*A*_ = 15, quantum efficiency is 60%, reconciliation efficiency is 0.967, the excess noise is 0.07 and the frame overhead is 10%.

Comparing to the results in theoretical regime, i.e., the infinite data block, a realistic block length limits the secure distance and one cannot remove this uncertainties completely. This is actually the finite-size effects which are mainly associated with the excess noise and privacy amplification. By making use of the realistic experiment conditions, we compute the final secret key rates according to Eq. (1). In our system, 50% of the optical pulses were used for generating the key and the rest were used for parameter estimation and frame overhead. Under this condition, the experiment and simulation results of the secret key rates are plotted in Fig. 3. The dash line (blue) represents the asymptotic theoretical key rate, and the solid line (red) denotes the computed theoretical key rate under the finite-size with block size of 10^9^. Theoretically, we can get a final secret key of 58.53 kbps at 50 km and 464.8 kbps at 30 km with the block size of 10^9^. However, due to the frame overhead for synchronization and other overhead, only about 90% data of the block size may be used for the secret key distillation and parameter estimation. Accordingly, the experiment result is about 52 kbps at 50 km which is expressed using cross in Fig. 3. To compare with the previous experiments, the results presented in refs [25,^28^,^30^] are also plotted. As a remark, we note that the theoretical value under the finite-size effect with sample block of 10^9^ is 58.53 kbps, while the experiment result is 52 kbps as mentioned above. This is due to the additional consumption which are mainly from the stability of the system, frame overhead, the precision of phase estimation and adjustment, and the interaction between Alice and Bob. These factors will be further optimized in the further investigation.

**Figure 4:**
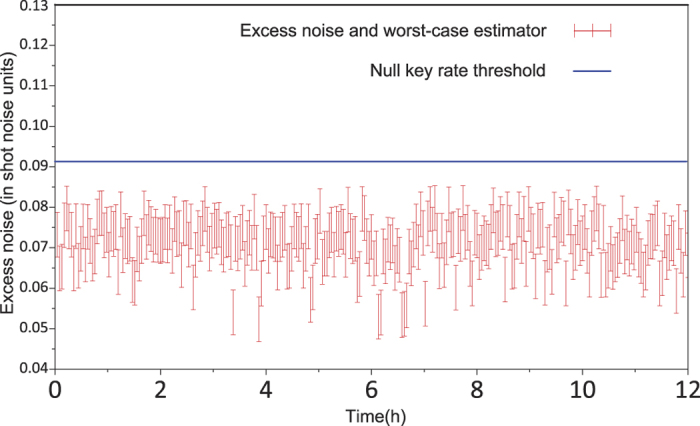
Experimental excess noise measured over 12 hours with a SNR of 0.9 on Bob’ side. In experiment, the length of fiber is 50 km corresponding to 10 db losses, the red pulse symbols indicate the measured excess noise and worst-case estimator, the size of block is 10^9^, which corresponding about 2.6 min of data sampling. The blue line shows the threshold of excess noise that can yield positive secret key rate.

The fluctuation of excess noise is an important issues. To confirm the impact of uncertainty of the excess noise on the secret key rate in real time, we measured the excess noise on the block with size of 10^9^ over 12 hours at a distance of 50 km, and calculated the key rate in this situation. We use the standard procedure for the excess noise estimation^35^, and estimate the parameters by randomly sampling *m* = *N* − *n* couples of correlated variables for getting the excess noise. Considering a calibrated shot noise variance 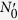, we can achieve a real-time excess noise in Alice’s side as 
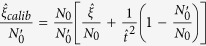
. The result is plotted in Fig. 4. We anticipate that the CVQKD with our technologies should remain effective for longer transmission distance by further controlling the excess noises in the practical CVQKD system.

## The Conclusion

In this paper, a practical CVQKD system is developed and it can run at 25 MHz repetition rate over 50 km optical fibre. A final secret key rate of 52 kbps at the distance of 50 km is obtained experimentally. Our system can stably work and meet the real-world conditions. Moreover, the performance of our CVQKD system can be further improved by involving higher speed homodyne detection and suppression of excess noise from instability and other defects of modules. Therefore, achieving a higher secret key rate is feasible by some direct improvements on our CVQKD system.

## Methods

### Polarization Feedback Algorithm

The proposed algorithm executes the following steps. A polarization feedback signal is produced by an amplified Root Mean Square to Direct Current (RMS-DC) conversion by picking out a 10% portion of the LO light in real time. This operation gives a set of normalized voltages denoted by *V*_*pola*_ = {*V*_*t*1_, *V*_*t*2_, …, *V*_*tm*_}. By making use of these voltage values, one may evaluate the mean value *μ*_*pola*_ and the standard deviation *σ*_*pola*_ of the polarization drift, i.e.,


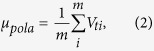



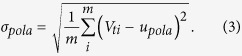


Then the jitter ratio of the polarization is calculated by,


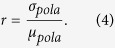


According to the jitter ratio, a dynamic polarization controller (DPC) on Bob’s side is employed to adjust automatically the polarization status in real time to maximize the LO power.

### Phase Compensation Algorithm

Denote the continuous variable sent by Alice as 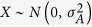, which can be expressed as,


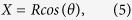


where *R* and *θ* are independent random variables, satisfying the Rayleigh distribution and the uniform distribution, respectively. Suppose the phase drift as Δ*ϕ* and the excess noise as 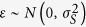, then the continuous variable *Y* received by Bob could be expressed as,


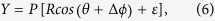


where the Δ*ϕ* is the phase drift and *P* is the proportionality factor considering the total transmission. We estimate the value of Δ*ϕ* and *P* by inserting one frame of test data *X*_*phase*_ into the sent data. Notice that both Alice and Bob know exactly *X*_*phase*_ in advance, and the testing frame will not be used for the key distillation. When Bob receives the data, he firstly extract the testing frame data *Y*_*phase*_ and then calculate the expectations of *X*_*phase*_ · *Y*_*phase*_. Then a proper phase bias Δ*θ*′ can be added into *X*_*phase*_ to generate 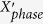 and calculate the expectations of 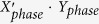. This gives an evaluated value of Δ*ϕ* and *P*. Finally, Alice reconstructs the data, i.e.,


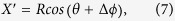


and Bob performs linear operation on the received data to eliminate *P* and obtains


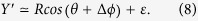


### Multidimensional reconciliation

In order to achieve higher reconciliation efficiency and speed, we implemented the multidimensional reconciliation with optimized decoder. In the multidimensional reconciliation scheme, for instance an 8 dimension case with reverse reconciliation scheme^36^, Bob divides his data *y* into blocks with size 8 and generates a binary random vector *u* with the same size. Then Bob computes vector 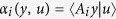, where *A*_*i*_ is a member of 8 orthogonal matrices, see^29^. After that, Bob sends *α*_*i*_ to Alice who draws function *M*,


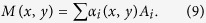


Making use of the obtained *M*, Alice calculates *v* = *M* · *x* which can be regarded as a noisy version of *u*. With a followed error correction stage, Alice and Bob can share a string of secret key.

On the other hand, higher speed reconciliation is achieved by reducing the complexity of the decoder for error-correction. Two methods, named as vectorization and parallelization, are implemented to improve decoding speed. Vectorization diminishes unnecessary calculations and saves lots of memory, while parallelization can further improve the efficiency of decoder after vectorization.

For example, one of the calculations involved in the decoding algorithm (the Belief-Propagation decoding algorithm) is


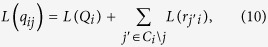


where *L*(*q*_*ij*_) and *L*(*r*_*ji*_) are the information passed between variable nodes and check nodes, and *L*(*Q*_*i*_) is the initial decoding information. The vectorization reduces variables *L*(*q*_*ij*_) and *L*(*r*_*ji*_) to 1-dimensional *L*(*q*_*n*_) and *L*(*r*_*n*_) thus reduces complexity,


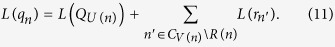


The parallelization can further reduce the complexity by paralleling frames together,


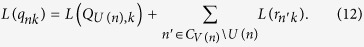


In this case, summation complexity remains the same while more frames are disposed. As a result, the overall complexity for decoding *K* frames are reduced so that the decoder is speeded.

In conclusion, the size of variables *q*_*ij*_ and *r*_*j*′*i*_ in Eq. (10) is reduced from *M* × *N* to *L*, where *L* is the number of element 1 in H, *M* and *N* are column and row number of H matrix respectively, and they generally satisfy *L*^2^/*N* < 0.01(*M* × *N*) in Eq. (11). In our case, *L* = 32400, *M* = 9800, *N* = 10000 and *L*^2^/*N* ≈ 0.001 * *M* * *N*. Furthermore, in Eq. (12), the efficiency of decoder can be further improved by reducing non-mathematical operations like function calling and loop control.

## Additional Information

**How to cite this article**: Wang, C. *et al.* 25 MHz clock continuous-variable quantum key distribution system over 50 km fiber channel. *Sci. Rep.* **5**, 14607; doi: 10.1038/srep14607 (2015).
